# Group Normalization for Genomic Data

**DOI:** 10.1371/journal.pone.0038695

**Published:** 2012-08-13

**Authors:** Mahmoud Ghandi, Michael A. Beer

**Affiliations:** McKusick-Nathans Institute of Genetic Medicine and the Department of Biomedical Engineering, Johns Hopkins University, Baltimore, Maryland, United States of America; University of Turin, Italy

## Abstract

Data normalization is a crucial preliminary step in analyzing genomic datasets. The goal of normalization is to remove global variation to make readings across different experiments comparable. In addition, most genomic loci have non-uniform sensitivity to any given assay because of variation in local sequence properties. In microarray experiments, this non-uniform sensitivity is due to different DNA hybridization and cross-hybridization efficiencies, known as the probe effect. In this paper we introduce a new scheme, called Group Normalization (GN), to remove both global and local biases in one integrated step, whereby we determine the normalized probe signal by finding a set of reference probes with similar responses. Compared to conventional normalization methods such as Quantile normalization and physically motivated probe effect models, our proposed method is general in the sense that it does not require the assumption that the underlying signal distribution be identical for the treatment and control, and is flexible enough to correct for nonlinear and higher order probe effects. The Group Normalization algorithm is computationally efficient and easy to implement. We also describe a variant of the Group Normalization algorithm, called Cross Normalization, which efficiently amplifies biologically relevant differences between any two genomic datasets.

## Introduction

**Figure 1:**
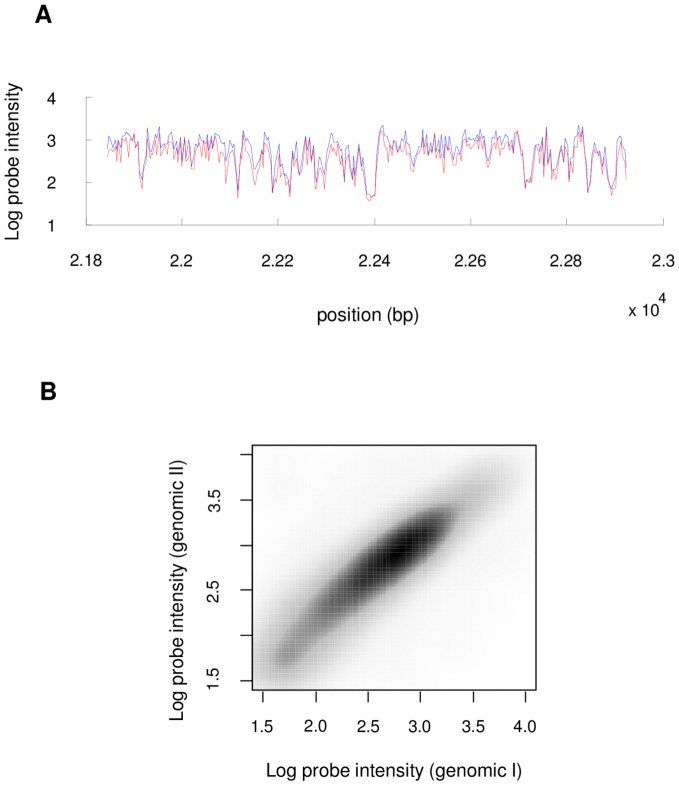
Genomic assays are often highly reproducible, but have significant efficiency variation across the genome. (A) Two genomic hybridization signals (biological replicates) from (Lee et al., 2007) shown along a portion of Chr III are highly reproducible, but deviate significantly from the expected constant signal. (B) Across the whole genome, these variations are highly reproducible. Two genomic hybridizations for the entire yeast genome are highly correlated (Pearson C = 0.966).

Recent advances in high-throughput technologies such as massively parallel sequencing and microarrays have allowed contemporary biological experiments to routinely measure changes in molecular binding or transcription throughout the genome. These experiments generate large scale genomic datasets whose accurate interpretation requires a preliminary normalization step. For example, DNA-protein interactions are commonly measured by quantifying the amount of isolated labeled target DNA by hybridization to complementary oligonucleotide probes on high density tiling microarrays (ChIP-chip), or by sequencing (ChIP-seq). In ref. [1], Affymetrix tiling arrays consisting of over 2 million oligonucleotides of 25 base pairs (bp) each, have been used to evaluate the genome wide nucleosome positioning in yeast. Our interest in the normalization problem arose from our analysis of this data. We noticed that the tiling array signal is generally highly reproducible; however, it suffers from high probe to probe variation. This is most strikingly shown when randomly sheared genomic DNA is hybridized to a tiling microarray (as a control). In this case, one expects to see a flat, or uniform, hybridization signal, as genomic DNA and tiling probes are present in equal amounts. In this experiment, shown in Figure 1, however, the signal deviates significantly from the expected flat curve, but is highly reproducible. Figure 1 shows the probe level signal for two independent genomic DNA hybridizations from ref. [1]. Similar results for ChIP-seq occur because of sequence dependencies of the sequencing assay, as shown in Figure 2a of ref. [2]. For clarity of presentation, we will focus our description on the case of microarray hybridization, and we will refer to the phenomenon of sequence specific assay efficiency as the *probe effect,* which causes probes with identical input DNA concentration to display differential hybridization intensity. This behavior can partially be explained by the fact that individual probes bind their target DNA with varying hybridization efficiency (for example because of their different GC content), but non-specific binding (NSB) and cross hybridization also contribute to differences in observed probe signals. In this paper we propose a novel method to infer the normalized input DNA levels from microarray data and correct for these probe effects. Most other genomic data sets involve similar probe or target sequence dependencies on assay sensitivity. For example, the sensitivity of massively parallel sequencing in a ChIP-seq or RNA-seq experiment might depend on the GC content at the 5′ end of the sequence. In addition to varying sequencing or hybridization efficiencies, the DNase or MNase assays used to prepare DNA may have subtle sequence affinity biases. While our method was developed by our interest in modeling Nucleosome positioning data, which will be the primary example throughout the paper, a similar approach could be applied to other genomic datasets. For example, detecting differential genomic binding of TFs due to natural variation [3] or differential gene expression from RNA-seq data [4] could benefit from our nonparametric normalization method. Improving the signal quality in genomic datasets strongly affects the accuracy and consistency of predictive models trained on this data (e.g. ref. [5]).

Another consideration is that most genomic experiments involve several conditions and/or replicates. There are usually at least two conditions: treatment and control, and for each condition there may be one or multiple replicates. Different arrays might have slightly different global experimental biases (due to image scanning, variable concentrations, etc.) which are conventionally removed by a global *normalization* using one of the several available methods (see ref. [6] for a comparison of different methods). After global normalization, the data are then *corrected for probe effect*.

Each of the normalization and probe effect correction algorithms makes assumptions about the distribution of probe signals that limits their general application. Quantile normalization [7], imposes identical probe signal distributions across conditions. This is achieved by replacing the probe signal in each condition by the mean value of probes at the same rank. Hence after quantile normalization all conditions will have the same histogram of probe signal and the Quantile-Quantile Plot (Q-Q Plot) will be a straight line. Although the assumption of identical distributions roughly holds in many cases, there are cases where it is clearly inappropriate. For example in case of nucleosome positioning, about 75–80% of the genome is nucleosome bound; hence the distribution of the probe values for a nucleosome enriched condition should be significantly different than that of the genomic control.

The rank-invariant set method [8], selects a set of probes with similar rank in all conditions. This set of probes identifies regions that do not vary significantly across the different microarray conditions; for example, they may be housekeeping genes whose expression levels vary only slightly in different conditions. A nonlinear model (e.g., splines) is then fitted to the variation among the invariant set probes and used to normalize the value of all other probes. Although the assumption of invariant activity of housekeeping genes may be valid in certain cases, in many other cases it can be difficult to define a suitable invariant probe set [9], [10]. This limits the application of this normalization method.

In more recent work, Sun, et al. [11] have used a mixture model approach for ChIP-chip analysis that uses LOESS curve fitting for normalization. They perform separate LOESS fitting for probes with similar GC content to better model the nonlinear relationship between probe signal on different arrays. Although this method gives promising results in a variety of ChIP-chip experiments, the strength of their method lies in its robust estimation of the null distribution. The validity of this approach is based on the assumption that the majority of probes are null, which while often the case, is not valid for nucleosome positioning data where over 70% of the genome is nucleosome enriched. Moreover, relying only on GC content limits the flexibility of their normalization algorithm.

After performing a global condition-to-condition normalization, it is important to account for the probe effect. Earlier Affymetrix array designs attempted to correct for the probe effect by estimating non-specific binding signal (NSB), and by controlling for efficiency by choosing probes with constrained GC content. To estimate the amount of the non-specific binding signal (NSB), on some Affymetrix microarrays, there exists a mismatch probe (MM) for each perfect match probe (PM). The MM sequence is identical to that of the PM with the exception of the central base which is complementary to the central base of the PM probe. Although having a MM probe for each PM probe can help to estimate the NSB signal in some cases, in many cases MM probes does not give a direct measure of the NSB and their successful use is limited in practice. Usually, for a significant fraction of probes, the MM signal is even higher than the PM, which may be caused by the different hybridization efficiencies of the PM and MM probes. The distribution of probe signals for PM and MM probes are shown in Figure S1 for the data in ref. [1], showing that they have significantly different distributions for the nucleosome enriched and genomic control conditions.

Since probe efficiency is mainly dependent on the sequence of the probes, some authors have proposed methods to directly estimate the hybridization signal (both the NSB and also the gene specific signal (GSB)) from the probe sequence [12], [13]. In ref. [12], the binding energies are approximated from a position dependent weighted summation of the dinucleotide stacking energies, and least squares fitting algorithms are used to estimate the parameters. In ref. [13], a sequence-based model for the probe affinity, called MAT, is proposed that includes a position dependent weight for each nucleotide. It also includes a nonlinear term proportional to the count of each nucleotide. It is shown that MAT can be effectively used to capture most of the probe to probe variability [13], however, even after MAT correction, the probe effect is not completely removed [14]. The reason for this is that MAT is not comprehensive enough to fully model the probe dependent effects which are known to be significantly nonlinear. More recent work [14] has proposed a new method called TileProbe, that employs publicly available data from the GEO database [15] to further remove the probe effect from MAT corrected intensities. TileProbe uses the median of the MAT corrected probe intensities over all samples as a model for the magnitude of the residual probe effect. Similar to ref. [11], it relies on the assumption that most of the probe signals are generated by the null distribution. This limits the application of this method for nucleosome positioning data, where most probes have signal. Moreover, although the above mentioned sequence-based models are based on physical quantities such as stacking energies, the actual parameters are obtained by curve fitting and least square optimization, which may lead to values that are not consistent with the original model. For example in Zhang's model [12], in some cases, the coefficients for some dinucleotides are estimated to be positive for NSB, but negative for GSB; further evidence that the sequence-based model is overconstrained.

A comprehensive model that can explain all of the probe variations at different conditions (DNA concentrations and temperatures) would be very complicated. In this paper, instead of using a model-based approach, we propose a data-based approach that integrates the normalization and probe effect correction steps and eliminates the need for an explicit underlying hybridization model. Instead, we estimate the parameters of a probe's response from the response of a similar set of probes. The proposed method has the advantage of being robust and simple (no curve fitting to estimate parameters) and can effectively be used to normalize probe values. The proposed method can also be used for microarray data at two different experimental conditions to differentially amplify regions that have changed from one condition to another. We demonstrate that this method can clearly and effectively identify the biologically relevant regions in the genome which respond to an experimental stimulus, and that these regions are frequently difficult to distinguish from noise using alternative approaches.

## Methods

### Group Normalization

We model the observed signal *y_i_* for a given probe *i* as linear combination of three terms: first, the signal *y_i_* is proportional to the desired biological signal, *x_i_*, with a probe specific efficiency, *A_i_*. Second, each probe has a background signal independent of *x_i_* which we model as a constant signal, *B_i_* (a combination of non-specific binding and other target independent signals), and a contribution from random noise, 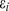.
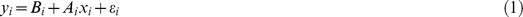
The normalized desired biological signal *x_i_* is unitless, and can be scaled arbitrarily. In the case of nucleosome positioning, we will use *x_i_* = 0 for fully unbound regions and 1 for fully nucleosome bound regions. The random noise, 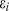, represents all factors that cannot be modeled by *A_i_* and *B_i_*. The goal of normalization is to determine *x_i_* from the observed signal *y_i_*, and we do so by estimating *A_i_* and *B_i_* for each probe. Although we will focus on tiling array signals for nucleosome positioning as an example, the model given in (1) is quite general and the normalization scheme proposed in this paper can be straightforwardly adapted to a variety of genomic assays, including Chip-seq.

**Figure 2:**
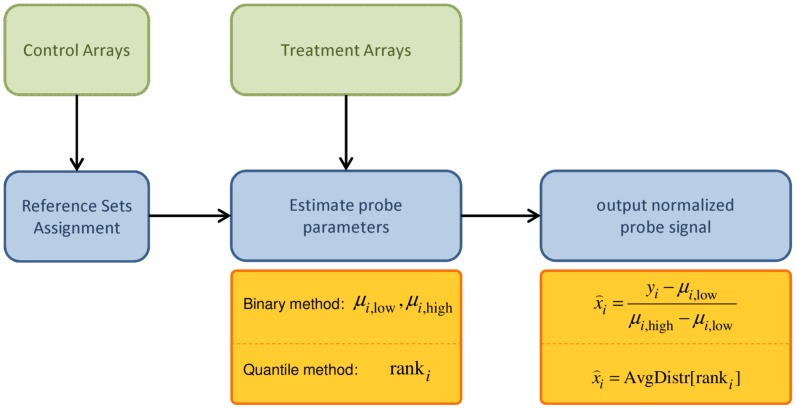
Flowchart of Group Normalization. Control arrays are used to generate reference probe sets for each probe. Then we use the reference probe sets to estimate the probe parameters in the treatment arrays and to generate the normalized signal. We propose two distinct methods to normalize the arrays: a Binary method which parameterizes high and low signal for each probe (μ_low_, μ_high_); or a Quantile-based method which uses the rank of each probe in the reference set.

**Figure 3:**
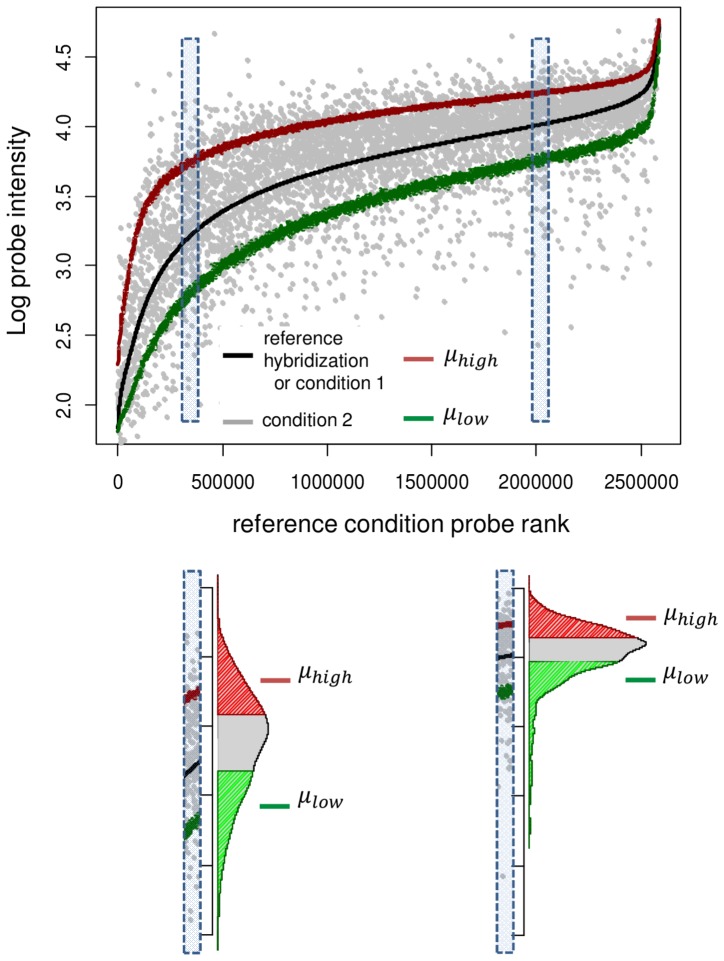
Overview of Group Normalization. Probes are shown sorted by on their values in a genomic hybridization (reference condition, black). For each probe, *N* = 1000 probes with closest signal in the genomic hybridization are assigned as reference set (dashed boxes) for each probe. Then high (red) and low (green) signal levels in the experimental condition (grey) are estimated from high and low probe signal ranges for each set of reference probes.

As briefly discussed in Introduction, the relation between the probe effect and the probe sequence is a nonlinear and relatively complicated relation. Instead of trying to develop a physically motivated model of this relation, in our approach, Group Normalization, we model this relation implicitly from the data. This is in contrast to most model based approaches, where an explicit model is assumed, and the model parameters are estimated by fitting to the data (e.g. [12], [13]). Our method relies on the fact that on each high density microarray, there exist a very large number of probes (in a typical Affymetrix oligonucleotide tiling array, there are more than 2 million probes). Figure 2, shows a flowchart of the proposed method. In this method, for each probe *p_i_*, we find a set of reference probes, denoted Ref(*p_i_*), that have similar probe effects to *p_i_* (i.e. for all the probes *p_j_* in Ref(*p_i_*), *A_j_* is similar to *A_i_* and *B_j_* is similar to *B_i_*). The key to our Group Normalization is a ranking method to define such a reference probe set. If Ref(*p_i_*) is large enough, (we typically use *N* = 1000 probes for the reference set) despite random variation in individual probes, the probe dynamic parameters *A_i_* and *B_i_* can be robustly estimated from the reference set probe intensities. Figure 3 outlines this idea for Group Normalization for a single reference condition; below we will also consider the case of multiple reference conditions. In Figure 3 we highlight this process for two probes, one with high signal and one with low signal, but the procedure illustrated here is applied to all probes. First, all probes are sorted by their intensity in a reference condition, e.g., a genomic DNA reference hybridization. For each probe *i,* the 1000 probes with similar rank in the reference condition define the reference set Ref(*p_i_*), shown in light blue. Then the reference set probes are sorted again by their value in a second condition, the experimental condition, e.g., nucleosome bound DNA. After sorting, the 1000 reference set probes define a mean for low signal probes, *μ_i,low_*, and high signal probes, *μ_i,high_*, within this reference set. The range of probe ranks which will define *μ_i,low_* and *μ_i,high_* are parameters chosen to be appropriate for the given application. In the case shown in Figure 3, we use the 30% lowest and highest ranking probes to define *μ_i,low_* and *μ_i,high_*, i.e., ranks 1–300 define low signal probes and 701–1000 define high signal probes within the reference set. Finally, the normalized probe value in the experimental condition is given by:
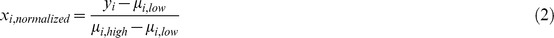
Our final results are insensitive to the definition of the high and low probe ranges. The simple procedure in Eq. (2) estimates the dynamic parameters *A_i_* and *B_i_* through *μ_i,low_* and *μ_i,high_*, and explicit values for *A_i_* and *B_i_* are not directly required. The basic idea is that within each reference set, there are probes with high signal in the experimental condition, and there are probes with low signal in the experimental condition, and these probes effectively determine *A_i_* and *B_i_* for the probe *p_i_*. We refer to the method explained above as the binary group normalization method since we estimate two signal levels (high and low) for each probe. In the following we explain an alternative approach, that does not involve estimation of *μ_i,low_* and *μ_i,high_*.

Instead of specifying a range of probes to determine *μ_i,low_* and *μ_i,high_*, a related approach would be to assume that the reference probes have the same distribution of the biological signal *x_i_*. Instead of defining the ranges for low and high probes, we apply quantile normalization, and use the signal in the quantile normalized average distribution as the normalized signal. In other words, we take the average of the reference set distribution for all the probes to find an average reference set distribution, then for each probe, the rank in the reference set is calculated and the normalized signal would be the value of the average reference set distribution corresponding to that rank. We have implemented this more general approach, which we refer to as quantile-based group normalization. Compared to binary group normalization, this method gives slightly better results with spike-in ChIP-chip dataset, but we get less signal to noise improvement in nucleosome positioning data.

### Copy number variation

In the definition of the reference set, we are implicitly assuming that all regions of the genome are responding to the same input DNA. But probes within repetitive regions may be responding to multiple copies of identical DNA throughout the genome, as shown in Figure S2 for the response of a region containing a Ty1 transposon in yeast to a genomic hybridization. Therefore, for purposes of defining the reference set probes, we explicitly ignore repeats, as these regions could reduce the accuracy of our estimation of the probe parameters.

### Reference group assignment

Group Normalization is based on finding a set of reference probes that have similar probe effects for each probe. In principle, this set could be found with high precision and provided by the chip manufacturer. But in practice, each laboratory's hybridization protocols might be applied under somewhat different conditions, and probes that have similar dynamic parameters in one lab might have different dynamic parameters in another lab. Or more generally, a good locally linear estimation of the probe parameters under some conditions might not apply to all conditions. We therefore recommend that individual users use a control ‘reference condition’, as described above, or a set of control ‘reference conditions’. Here we propose three methods to find such reference sets: 1) a single reference method, 2) a sorted average method for multiple reference conditions, and 3) a minimum distance method for multiple reference conditions.

1- Single reference method:

In this approach we perform one genomic hybridization experiment (the reference condition), as described above. In this experiment, every probe should measure an equal amount of DNA. However, since oligonucleotide probes have different affinities and dynamic properties, the measured signal is not uniform (see Figure 1). To define the reference set we simply sort all the probes based on their value in the reference condition and for each probe we assign the *N* neighboring probes as the reference set (Figure 3).

2- Sorted average method.

Since any single hybridization is susceptible to some amount of random noise, using multiple reference hybridization may provide a more robust estimation of an appropriate reference set. When multiple reference hybridizations (conditions) are available, we propose using the sorted average method, where we sort all probes by their average signal in multiple reference hybridizations. We then assign *N* neighboring probes as the reference set for each probe.

3- Minimum distance method.

The sorted average method minimizes the bias in estimation, but by only using the average signal, we are losing data about the variance of the probe values in the multiple reference conditions, which also gives us information about a probes' reliability and our confidence in our estimation of its parameters. To improve the reference set assignment, the minimum distance method selects *N* nearest probes in the multidimensional reference condition space. For example, say we have performed *M* genomic control hybridizations, then the distance between two probes *i* and *j* in *M-*dimensions is defined to be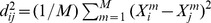, where 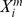
is the *i*'th probe signal in the *m*'th reference condition. So for each probe *p_i_*, we assign the *N* probes with minimum distance to *p_i_* to the reference set. This way, the probability that a highly variable probe (unstable from dataset to dataset) might mistakenly be assigned as a reference probe is decreased. Figure S3 depicts an example where the sorted average method and min distance methods give different results.

### Cross Normalization

In many applications, we are interested in detecting significant differences between two conditions, neither of which is a genomic control. For example, we may be interested in comparing the early and late response to a stimulus, comparing pre- and post-stimulus, or comparing the response to two different stimuli. If a genomic hybridization is available, we could separately normalize pre- and post-stimulus to the genomic control, as described above, and then compare the two normalized signals. However, we are really interested in detecting changes between the two conditions, not changes relative to the genomic control. In this case, a more sensitive method to detect the relevant changes between the two conditions would be to directly apply the normalization algorithm described above to the two conditions, i.e. use the pre-stimulus data as the reference condition for the post-stimulus data, and use the post-stimulus data as the reference condition for the pre-stimulus data. This amplifies the differences between the two signals. Then the correspondence between these asymmetric approaches is a measure of the reliability and significance of the detected changes in normalized signal.

### Signal Quality Measurement

**Figure 4:**
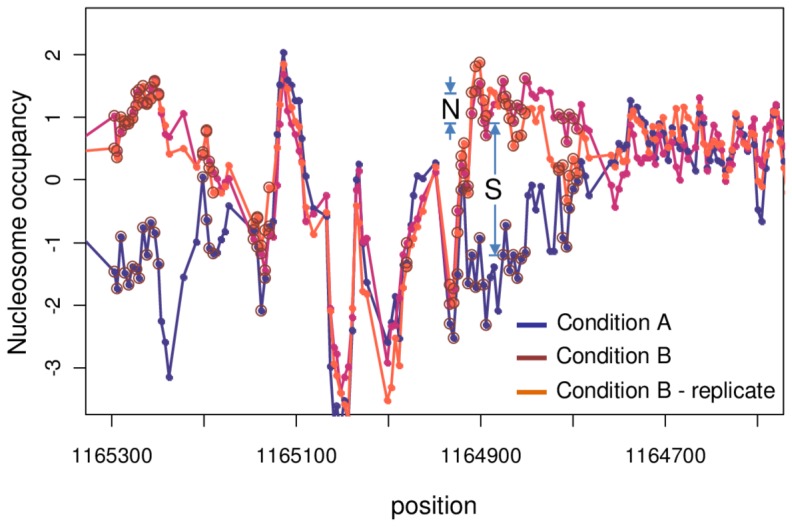
Signal Quality measure. Two tiling array signals corresponding to nucleosome occupancy at two different experimental conditions are shown for the *HXT3* locus. We use two conditions and a replicate to determine signal and noise, as follows. In condition A (with glucose), the highlighted region is nucleosome free, and in condition B (no glucose), it is nucleosome bound. *S* is the difference of the tiling array signal at two different conditions and reflects the signal strength. *N* is a measure of noise and is estimated by comparing the signal of two replicate microarrays at similar experimental condition. We evaluate *S* over a set of significantly changed probes (indicated with open circles) and *N* over all the probes as described in the text. The ratio *S/N* is a genome wide measure of Signal Quality.

In the following, we describe a method to quantitatively evaluate the performance of the proposed normalization methods. We define the Signal Quality measure as shown in Figure 4. We assume that biologically significant changes between any two arrays will have significantly different signal, but will also have a spatial extent that covers many adjacent probes. To define a set of probes whose signal is significantly changed in two conditions, we use the top 2% of probes sorted by the difference in signal between the two arrays spatially averaged over a window of 147 bp. To avoid biasing the results toward one normalization approach, we use the intersection of the top 2% probes from each approach being compared (say, Group Normalization, Affymetrix MAS5 [16], MAT [13], and quantile normalization [7] of raw data, for which there was a 61% overlap between the top 2% probes using the four methods). These significantly changed probes are indicated by open circles in Figure 4. Then the signal power, *S*, is defined to be the mean square change of the signal on these probes between conditions A and B. The noise power, *N*, is the mean square change of the signal on entire probes between condition B and a replicate of condition B, as depicted in Figure 4.

Using data from [17], three tiling arrays at *T* = 0 (prior to glucose addition) and three tiling arrays at *T* = 60 mins (after glucose addition) were independently normalized against four separate genomic controls using Group Normalization (binary method with four sets of low and high probe ranges as described above, and quantile based method), MAS5, MAT, and quantile normalization of raw data. To make a fair comparison between MAS5 and the other methods, we used a running average window of 20 bp to match the 20 bp bandwidth in MAS5. Then the Signal Quality, *S*/*N*, over the significantly changed probe set in dB was calculated as 10*Log_10_(*S*/*N*), for all 36 combinations of conditions A, B and replicates. We use these combinations to estimate the mean and standard deviation of the improvement in Signal Quality.

### Detecting enriched regions in a Spike-in Benchmark dataset

To compare the performance of the group normalization, with some other existing methods, we applied this method on the Affymetrix and Agilent arrays data in the benchmark spike-in dataset [18]. For Affymetrix arrays, among the methods compared in [18] MAT gives the best performance. To compare our method with MAT, we substituted the probe standardization step in MAT with group normalization and used the same method to detect enriched regions. We used rMAT [19] with the following parameters: dMax = 600, dMerge = 300, nProbesMin = 8, method = “pValue”, threshold = 0.0001. For Agilent arrays, among the methods compared in [18] Splitter gives the best performance. To compare our method with Splitter, we substituted the normalization step in Splitter with group normalization and used the same method to detect enriched regions. We used the online implementation of Splitter (http://zlab.bu.edu/splitter) with the following parameters: maxgap = 200, minrun = 2, mean, Signal cutoff = 2.5s.d.

To compare the performance of the methods, we plotted the ROC-like curves similar to [18] and used area under the ROC-like curves as a measure to compare different methods. For a perfect classifier this area is 1, and for random it is near zero.

## Results

**Figure 5:**
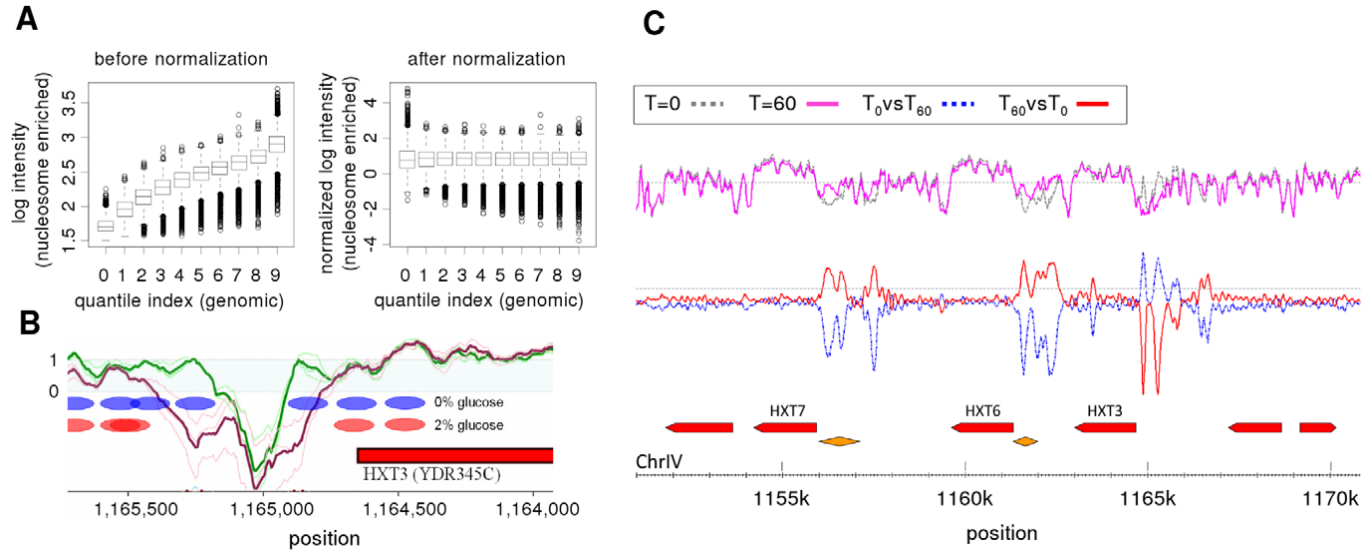
Group Normalization results for nucleosome positioning in yeast. (A) probe distribution before (left) and after (right) Group Normalization. (B) Inferred nucleosome pattern at HXT3 promoter before (blue ovals) and after (red ovals) glucose addition. HXT3 is upregulated at high glucose levels and repressed at low glucose levels. (C) Differential nucleosome occupancy in yeast in response to glucose addition: cells are grown on glycerol and then 2% glucose is added. Nucleosome positioning is measured before and 60 min after glucose addition (Zawadzki et al., 2009). The top curves show the spatially averaged raw tiling array data, at time zero (gray dotted) and t = 60 (magenta). The lower plot shows the result of our normalization method. The red curve is the normalized differential nucleosome occupancy for t = 60 min compared to t = 0 (high values imply increase in nucleosome occupancy in response to glucose). The blue dotted curve is the reverse analysis, comparing t = 0 to t = 60. The yellow diamonds indicate ADR1 binding regions from ChIP.

We applied the proposed Group Normalization method on published genome-wide nucleosome positioning data in yeast [1], [17], [20]. The joint distribution of probes in the experiment (nucleosome enriched tiling array) and control (genomic hybridization) before and after normalization is depicted in Figure 5A. Before normalization, there is a high correlation between signal in the experiment and in the control, which reflects the strong probe effect. This correlation between treatment and control is almost completely removed by our Group Normalization. Figure 5B shows normalization results for nucleosome occupancy near the HXT3 promoter before, and 60 minutes after, glucose addition. The array signal changes significantly between the two conditions, with a spatial scale of ∼150 bp, indicating that nucleosomes at the promoter are removed after glucose addition. Figure 5C shows the results for the cross normalization algorithm along the broader HXT locus on chromosome 4. The top row shows the 20 bp running average of the raw data and the bottom row shows the results using the cross normalization procedure described above. Cross normalization highlights the regions of differential nucleosome occupancy across this locus much more clearly than can be detected from the raw data. Specifically, it is clear from the cross normalization that while nucleosomes are depleted from HXT3 promoter upon glucose addition, new nucleosomes have been placed at HXT6 and HXT7 promoters. These changes are consistent with the expression level changes for these genes, and with the facts that HXT3 is a low affinity glucose transporter and its expression is up-regulated upon addition of glucose [21], and that HXT6 and HXT7 are high affinity glucose transporters.

**Figure 6:**
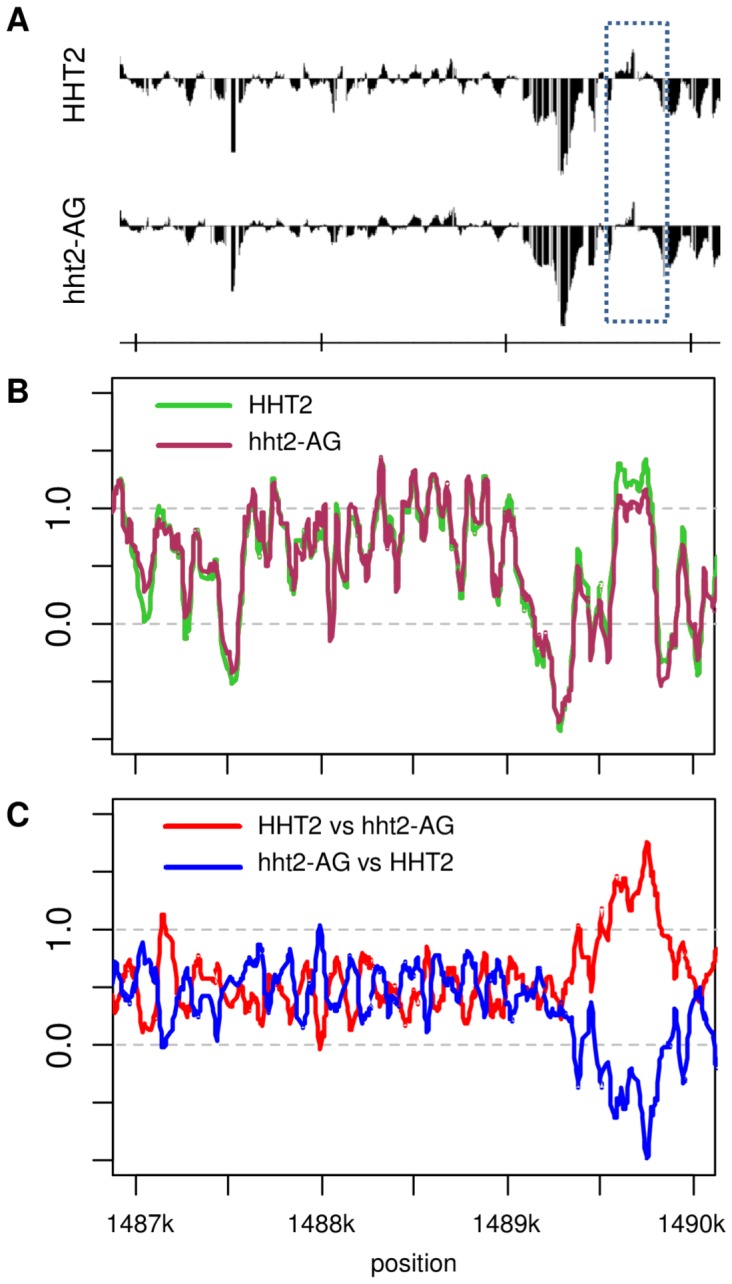
Group Normalization results for histone H3 mutant dataset. Nucleosome occupancy in wild type (HHT2) and histone H3 mutant (hht2-AG) near AGE1 on yeast chromosome IV is show for region plotted in Figure 8 of (He et al 2008). A) Nucleosome occupancy plots using Affymetrix TAS software as was used by (HE et al 2008). The dotted box shows the location for the change in nucleosome occupancy. (B) Group Normalization makes it somewhat easier to detect the differentially occupied promoter and clearly identifies the bound regions, but (C) cross normalization more strongly amplifies the differentially occupied region.

We next applied our Group Normalization method to another nucleosome occupancy dataset, measuring nucleosome occupancy in a histone H3 mutant strain [20]. Figure 6 depicts the results of [20], who used Affymetrix Tiling Analysis software (TAS) provided by Affymetrix. We reproduced the nucleosome occupancy profile in the AGE1 locus over the same region shown in Figure 8 of ref. [20], shown in Figure 6A. We also used Group Normalization to process the data for the same region, shown in

Figure 6B. The most significant change in nucleosome occupancy near AGE1 is that nucleosome binding has been reduced in the histone H3 mutant compared to wild type strain. While this is barely detectable in Figure 6A, and somewhat more evident with Group Normalization in Figure 6B, cross normalization differentially amplifies these differences in nucleosome occupancy in the two strains, as shown clearly in Figure 6C, highlighting the significant difference in occupancy upstream of AGE1.

**Figure 7:**
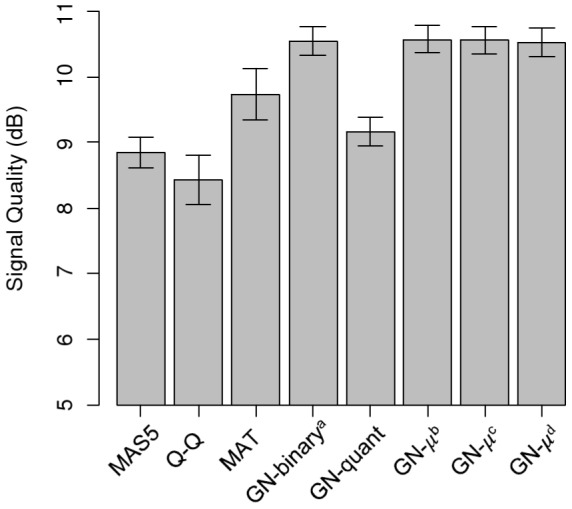
Signal Quality comparison of Group Normalization to other methods. We applied different normalization methods to the nucleosome positioning data and measured the Signal Quality using MAS5, quantile normalization (Q-Q), and MAT. Binary Group Normalization (GN-binary) has higher Signal Quality than all other approaches tested. Quantile normalization (GN-quant) outperforms MAS5 and Q-Q but not MAT on this dataset. We also examined the sensitivity of binary Group Normalization to different choices of low and high probe ranges used to estimate μ_low_ and μ_high_: (μ_low_, μ_high_) = a: (.10–.40,.60–.90), b: (.05–.50,.80–.95), c:(.10–.50,.50–.90), and d:(.10–.30,.70–.90). All of these choices give virtually identical Signal Quality improvement.

To quantitatively compare the genome-wide performance of the proposed normalization method with existing methods, we defined a Signal Quality measure (see Methods), the difference of the signal of two microarrays at two different experimental conditions (Signal) divided by difference of two microarrays at similar condition (Noise). Following refs. [1], [17], [20], who used the Affymetrix MAS5.0 algorithm to process their tiling array data, we compared our Group Normalization method with MAS5.0. We also computed the MAT [13] normalized signal and quantile normalized signal [7] for comparison. We measured the Signal Quality for regions of the genome that are differentially occupied before and after addition of 2% glucose [17] across the whole genome. As shown in Figure 7, Signal Quality was significantly improved from 8.8(0.7) dB using MAS5 to 10.5(0.6) dB using binary group normalization, showing 1.7(0.4) dB improvement in Signal Quality compared to MAS5.0 and 1.1(0.8)dB improvement compared to MAT. The numbers in parenthesis are the standard deviation for thirty six different data sets as explained in the methods section. Also as shown in this figure, using different ranges for low and high probe didn't result in a significant difference in binary group normalization method performance. When we used the alternative quantile-based group normalization method (see Methods) we found a Signal Quality of 9.2(0.6) dB, so the improvement is 0.4(0.3) dB compared to MAS5 but significantly less than binary method.

The performance of group normalization is not very sensitive to the definition of high and low probe ranges. Considering the probe signal model of Equation (1), if the reference set probes are independent of the biological signal, *x_i_*, then for all reference sets, the observed signal *y_i_*, would have a similar expected distribution to *x_i_*, scaled by *A_i_* and shifted by *B_i_*. Therefore, using different ranges for low and high signal, would give the same normalized signal, except for a constant shift and scale factor, which are functions of the ranges used for low and high signal estimation. To examine the sensitivity of the method to the choice of the low and high probe ranges, we used four different sets of ranges for low and high probes: a) 10%–40%, 60%–90%, b)5%–50%, 80%–95%, c)10%–50%, 50%–90%, d)10%–30%, 70%–90%, and compared the performance of the method using each set of ranges for the nucleosome positioning data, also shown in Figure 7. Since as expected, the performance was very similar for all the four different ranges, we only used one set of ranges, 10%–40% to define *μ_i,low_* and 60%–90% to define *μ_i,high_* for the spike-in ChIP-chip analysis.

**Figure 8:**
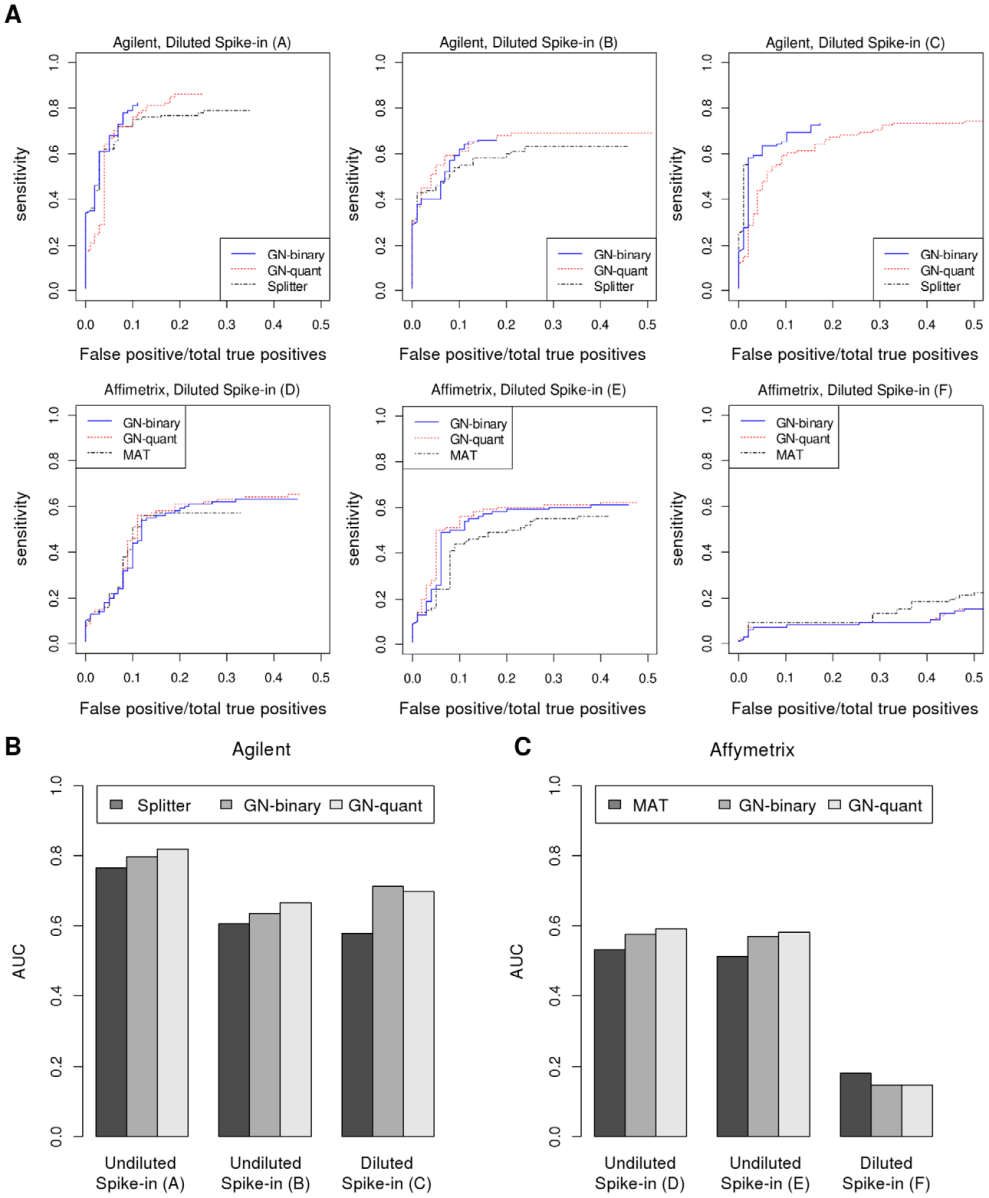
Comparison with spike-in benchmark data of Johnson et al. (2008). A) We compare ROC-like curves for different platforms and algorithms: Splitter, which had the best performance on Agilent data, and MAT, which had the best performance on Affymetrix data. Area under the ROC-like curve (AUC) is shown for B) Agilent and C) Affymetrix datasets. Except for the diluted Affymetrix spike-in data, which had poor performance with all methods, Group Normalization (both GN-binary and GN-quant) consistently performs better than previous methods, and has a higher sensitivity to recover spike-in regions at the same false positive rate.

To further evaluate our proposed method against existing methods we applied the group normalization method on the benchmark spike-in dataset [18]. In this dataset, a known amount of DNA from defined cloned regions was spiked in to genomic DNA and performance of different platforms and algorithms was assessed by comparing their ability to accurately recover the spike-in regions. Compared to nucleosome positioning data, where a significant fraction of the probes differ in treatment and control conditions, in this data set the spike in regions only cover about 0.2% of the probes, which is similar to expected ChIP-chip data with limited targets. We compare our method's ability to detect these spike-in regions to alternative methods in Figure 8. Group Normalization using either the binary method (GN-binary) or quantile method (GN-quant) detects more spike regions than Splitter or MAT at the same false positive rate, as shown by the ROC-like curves in Figure 8A, defined as in ref. [18]. The area under these curves summarizes the performance of each algorithm, as shown in Figure 8B and 8C. Except for the diluted Affymetrix dataset, which had low signal quality for all normalization methods, group normalization shows consistent improvement over previous approaches.

## Discussion

We have presented a new normalization procedure for genomic datasets. Our approach is based on the idea that genomic data sets have such a large number of sequences or probes that we can estimate the sequence biases of hybridization or sequencing (the dynamic parameters of the probes) from the response of probes across datasets. Because this approach estimates the dynamic parameters of a probe from a group of similar probes, we call our method Group Normalization. We have also described an approach based on this technique which highlights regions of significant signal changes between two experiments (cross normalization). We have shown that these normalization procedures can significantly improve the signal quality relative to existing normalization methods. We have shown that Group Normalization improves signal to noise in nucleosome positioning datasets and can more accurately identify spiked-in regions in the benchmark data of ref. [18] in most cases.

While signal quality is a global measure which shows the benefit of Group Normalization compared to other approaches, in some cases, the biology under consideration can also show how Group Normalization improves the analysis of the data. For nucleosome positioning, the known spatial scale of DNA bound to the nucleosome constrains the signal to vary on a scale of 150 bp. We can use genome wide nucleosome occupancy data to construct the autocorrelation of the nucleosome bound DNA signal for data that has been normalized using Group Normalization or other approaches. Because of nucleosome packing, we expect this autocorrelation function to exhibit periodicity on a scale set by the 150 bp nucleosome bound DNA plus linker DNA. As shown in Figure S4, the autocorrelation from group normalized data shows a stronger recurrence at 170 bp in the autocorrelation function compared to MAS5.

A possible extension of this model would incorporate an estimate of the variance of the probe signal from the reference set, and use that as a measure of the reliability of a probe. MAT uses a similar approach when combining the signal of neighboring probes.

While in this paper we have only presented results on tiling microarray data, in principle, other high-throughput technologies could benefit from a model-independent normalizationapproach similar to Group Normalization. Our development of the Group Normalization procedure was motivated by the observation that tiling array probes exhibit widely varying hybridization efficiencies, presumably due to non-uniform variations in the local sequence properties of the probes. We normalized these varying hybridization efficiencies by finding a large set of similarly responding probes in a reference condition. Most other genomic technologies suffer from analogous sequence specific effects on the assay efficiency. These could range from sequence dependent shearing rates, endonuclease sequence cleavage preferences, or sequence specific priming efficiencies in the case of massively parallel sequencing assays (RNA-seq or Chip-seq). Because we do not try to explicitly model the sequence specificity of the assay, but instead infer (estimate) sequence specific probe effects from a reference set of probes, our proposed approach should be useful in these cases as well.

## Supporting Information

Figure S1 **Mismatch probe distributions vary significantly in different conditions.** Histograms for one treatment (nucleosome enriched, top) and one control (genomic DNA, bottom) microarray are shown. The histogram for PM (left) and MM (right) probes are plotted separately.
(EPS)

Figure S2 **Repetitive elements have large variations in probe signal and are removed from the reference set computation.** Raw probe signals for genomic hybridization (control) near YBLWTy1-1 locus on chromosome II in yeast are shown.
(EPS)

Figure S3 **When multiple conditions are available, minimizing distance yields a more reliable reference set assignment.** Here for three different probes: p1 = (0.1,−0.1), p2 = (−0.3,0.3), p3 = (0.15,−0.20), the averages are Avg(p1) = 0; avg(p2) = 0; avg(p3) = −0.025; but distances are d12 = 0.4, d13 = 0.1. So although probe 1 and 2 have more similar averages, probe 1 and 3 have more similar responses, and probe 3 is therefore a better reference probe for probe 1.
(EPS)

Figure S4 **Comparison of autocorrelation of normalized nucleosome occupancy using Group Normalization and MAS5 algorithms for the (Lee et al 2007) data.** Group normalization shows a slightly higher recurrence in nucleosome occupancy signal due to the periodic packing of nucleosomes.
(EPS)
