# Supplementary figures and images for: Group Normalization for Genomic Data
Source: PLoS One. 2012 Aug 13;7(8):e38695. doi: 10.1371/journal.pone.0038695 (PMC3418286; doi:10.1371/journal.pone.0038695)

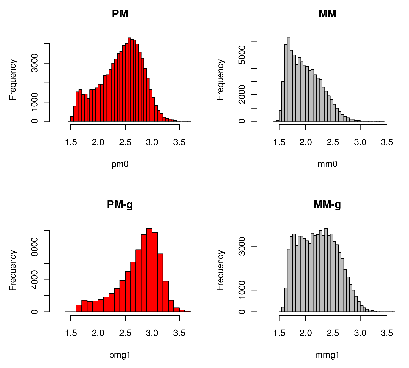

Supplement: Figure S1 — Mismatch probe distributions vary significantly in different conditions. Histograms for one treatment (nucleosome enriched, top) and one control (genomic DNA, bottom) microarray are shown. The histogram for PM (left) and MM (right) probes are plotted separately. (EPS) [file pone.0038695.s001.tif]

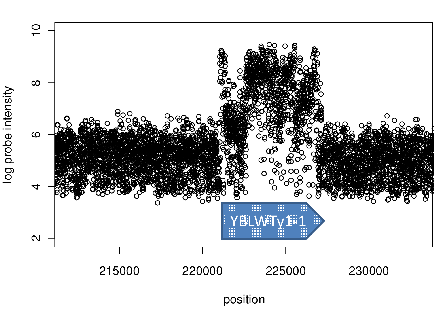

Supplement: Figure S2 — Repetitive elements have large variations in probe signal and are removed from the reference set computation. Raw probe signals for genomic hybridization (control) near YBLWTy1-1 locus on chromosome II in yeast are shown. (EPS) [file pone.0038695.s002.tif]

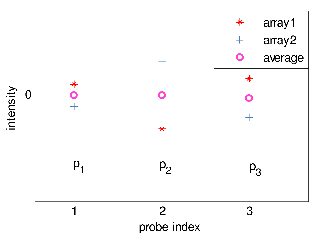

Supplement: Figure S3 — When multiple conditions are available, minimizing distance yields a more reliable reference set assignment. Here for three different probes: p1 = (0.1,−0.1), p2 = (−0.3,0.3), p3 = (0.15,−0.20), the averages are Avg(p1) = 0; avg(p2) = 0; avg(p3) = −0.025; but distances are d12 = 0.4, d13 = 0.1. So although probe 1 and 2 have more similar averages, probe 1 and 3 have more similar responses, and probe 3 is therefore a better reference probe for probe 1. (EPS) [file pone.0038695.s003.tif]

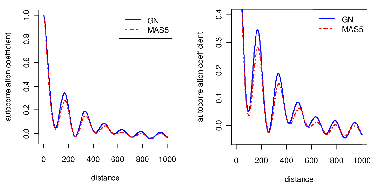

Supplement: Figure S4 — Comparison of autocorrelation of normalized nucleosome occupancy using Group Normalization and MAS5 algorithms for the (Lee et al 2007) data. Group normalization shows a slightly higher recurrence in nucleosome occupancy signal due to the periodic packing of nucleosomes. (EPS) [file pone.0038695.s004.tif]
